# Interpretable machine learning model for comparing and validating three diagnostic criteria for bronchopulmonary dysplasia in predicting value of respiratory prognosis of preterm infants: a retrospective cohort study

**DOI:** 10.3389/fped.2025.1678244

**Published:** 2025-11-25

**Authors:** Qiqi Bu, Xin Wang, Yanyan Wu, Yingyuan Wang, Rui Li, Yanmei Zhao, Caijun Wang, Guiying Sun, Wenqing Kang

**Affiliations:** 1 Department of Neonatal Intensive Care Unit, Zhengzhou Key Laboratory of Newborn Disease Research, Children’s Hospital Affiliated to Zhengzhou University, Zhengzhou, China; 2 Henan Provincial Clinical Medicine Research Center for Pediatric Diseases, Henan Key Laboratory of Pediatric Genetics and Developmental Diseases, Children’s Hospital Affiliated to Zhengzhou University, Zhengzhou, China

**Keywords:** bronchopulmonary dysplasia, respiratory prognosis, diagnostic criteria, machine learning, shap, validation

## Abstract

**Background:**

Comparison and validation of the predictive value of three diagnostic criteria for bronchopulmonary dysplasia for respiratory prognosis of preterm infants with gestational age <32 weeks.

**Methods:**

This retrospective cohort study was conducted to collect clinical data of 397 preterm infants. On the basis of the follow-up results, the enrolled population was divided into a respiratory adverse outcome group and a normal outcome group. The 2001 NICHD, the 2018 NICHD, and the 2019 NRN criteria were used to diagnose and grade BPD in preterm infants. The dataset was randomly divided, with 70% used for model training and 30% used for model validation. The extreme gradient boosting machine learning algorithm was used for model training. Furthermore, the SHapley additive exPlanation analysis method was utilized to visually interpret the results of the machine learning model.

**Results:**

A total of 397 preterm infants were included. In the training set, prediction models based on the 2001 NICHD, 2018 NICHD, and 2019 NRN criteria achieved AUC values of 0.747, 0.804, and 0.789, with corresponding accuracies of 0.740, 0.765, and 0.765. In the test set, the respective AUC values were 0.694, 0.747, and 0.752, and accuracies were 0.750, 0.800, and 0.750. Based on the DeLong's method, comparisons of ROC curves between the training and test sets revealed that both the 2018 NICHD and 2019 NRN criteria demonstrated significantly higher AUC than the 2001 NICHD criteria (training set: *Z* = −3.514, −2.110, both *P* < 0.05; test set: *Z* = −2.137, −2.199, both *P* < 0.05). However, there was no statistically significant difference in the AUC between the 2018 NICHD and 2019 NRN criteria for either the training set (*Z* = 0.863, *P* = 0.388) or the test set (*Z* = −0.176, *P* = 0.861). The SHAP revealing that the two most important features affecting the respiratory prognosis of preterm infants were the severity of BPD and early invasive ventilation.

**Conclusions:**

Both the 2018 NICHD and 2019 NRN criteria for BPD show better and similar predictive values for respiratory adverse outcomes in preterm infants, and both are superior to the 2001 NICHD criteria. The top two factors affecting the respiratory prognosis of preterm infants are the severity of BPD and early invasive mechanical ventilation.

## Background

Bronchopulmonary dysplasia (BPD) is one of the most common complications in preterm infants and develops from the combined effects of prenatal and postnatal factors on the basis of genetic factors (1). It is characterized by simplified alveolar and pulmonary vascular development and primarily occurs in preterm infants who require continuous oxygen therapy for more than 28 days after birth and remains oxygen dependent (2). Along with social progress, economic development, and medical improvement, the survival rates of premature infants have significantly increased (3). However, the incidence of BPD remains persistently high and has shown an increasing trend over time (4). According to a 2022 National Institute of Child Health and Human Development (NICHD) (5) report, the incidence rate of BPD during 2013–2018 was documented as 49.8%, representing a 5% increase compared to the previously reported 44.7% in their 2008–2012 cohort (6). BPD not only has a high incidence but also faces the problem of long-term adverse outcomes. Many infants with BPD experience suboptimal quality of survival after hospital discharge and still require multidisciplinary therapeutic management (7). A follow-up studies have shown that children with BPD are prone to recurrent wheezing and multiple rehospitalizations due to recurrent respiratory infections within 2–3 years after birth (8). Studies have indicated that under the diagnostic criteria of 2001 NICHD, 2018 NICHD, and 2019 NRN, the severity of BPD was associated with adverse respiratory outcomes, including neonatal complications and mortality, the use of respiratory medications during hospitalization, and the need for supplemental oxygen at discharge (9–11). Infants with BPD who experience adverse respiratory outcomes often exhibit dependence on mechanical ventilation and high-concentration oxygen, recurrent chronic hypoxic episodes, and extrauterine growth retardation. These patients typically require prolonged hospitalization and face high mortality rates. Long-term sequelae may include structural lung abnormalities and decreased pulmonary function, leading to recurrent respiratory infections, wheezing, chronic obstructive pulmonary disease, and other respiratory complications, ultimately contributing to a substantial disease burden (12). Thus, early diagnosis and monitoring of high-risk infants with BPD are crucial. Using appropriate diagnostic criteria to better predict adverse outcomes can facilitate early intervention and active treatment for high-risk infants, reduce severe complication rates, and maximize long-term prognosis improvement.

Machine learning is a technique in the field of artificial intelligence. Its essence is to enable computers to analyse and learn patterns from vast amounts of data, thereby enabling them to make predictions or decisions in new situations (13). In recent years, machine learning has gained significant attention in the medical field, as it has demonstrated remarkable ability in predicting clinical adverse events (14). It has been extensively applied across critical domains, including disease diagnosis, therapeutic intervention, and pharmaceutical research and development. Compared with traditional statistical methods, machine learning places greater emphasis on predictive accuracy and demonstrates the capability to identify patterns within high-dimensional datasets (15). Extreme gradient boosting (XGBoost), an ensemble learning model, aims to create a powerful model by combining multiple weak learners (typically decision trees). It has the advantages of short training time and high precision, and it balances performance and interpretability (16, 17). The SHapley Additive exPlanation (SHAP) is a *post-hoc* interpretability method that enhances the interpretability of machine learning classification models by quantifying each feature's contribution to classification outcomes through local or global computations, thereby elucidating the model's decision-making process (18).

This study aimed to combine XGBoost and SHAP to build an explainable framework for enhancing machine learning interpretability. It would compare and validate the predictive value of three BPD diagnostic criteria for adverse respiratory outcomes in preterm infants using machine learning methods, to guide the selection of clinical BPD diagnostic and staging standards.

## Materials and methods

### Study design and participants

This retrospective cohort study included infants with a gestational age (GA) of <32 weeks admitted within 28 days of birth to the Department of Neonatology of Children's Hospital Affiliated with Zhengzhou University between September 2021 and September 2023. The exclusion criteria were as follows: (1) severe congenital malformations, chromosomal defects or inherited metabolic disease; (2) death during hospitalization, transfer to another hospital, discharge against medical advice, or abandonment of treatment; and (3) incomplete data and loss to follow-up. This study has been approved by the Ethics Committee of the Children's Hospital Affiliated with Zhengzhou University (2024-KY-0084-001). All guardians/parents gave written informed consent.

### Data collection

This cohort study documented infant data, including sex, gestational age, birth weight, respiratory support status, weight at postmenstrual age (PMA) 36 weeks, pulmonary surfactant administration, hospitalization complications, length of hospital stay, and oxygen requirements at discharge. Maternal pregnancy-related data, including gestational complications, prenatal hormonal therapy, and pregnancy maintenance therapies, were also recorded. All the data were collected through the hospital's electronic health record system.

### Relevant diagnostic criteria

The diagnostic and staging criteria for BPD were as follows: (1) According to the 2001 NICHD criteria (19), preterm infants with a gestational age of less than 32 weeks who were treated with 21% oxygen for at least 28 days were diagnosed with BPD. The severity of BPD was classified as mild, moderate, or severe on the basis of whether the infant required oxygen at PMA for 36 weeks, the fraction of inspired oxygen (FiO2), and the mode of oxygen delivery (Table 1). (2) According to the 2018 NICHD definition (20), a diagnosis of BPD requires not only parenchymal lung disease with radiographic evidence but also respiratory support and FiO2, as shown in Table 1 for at least 3 consecutive days at 36 weeks' PMA to maintain arterial oxygen saturation at 90%–95%. (3) The 2019 Neonatal Research Network(NRN) (21) definition categorizes BPD severity solely on the basis of the mode of respiratory support at 36 weeks' PMA, regardless of supplemental oxygen use.(Table 1).

**Table 1:** Definitions of BPD.

Criteria	Grade	The mode of oxygen support			
2001 NICHD criteria	Mild BPD	Breathing room air at 36 wk PMA or discharge,whichever comes first			
	Moderate BPD	Need for 30% oxygen at 36 wk PMA or discharge, whichever comes first			
	Severe BPD	Need for 30% oxygen and/or positive pressure, (PPV or NCPAP) at 36 wk or discharge, whichever comes first			
2018 NICHD criteria		IMV	NCPAP, NIPPV, or nasal cannula ≥ 3 L/min	Nasal cannula (1∼<3) L/min or hood O2	Nasal cannula < 1 L/min
	I BPD	-	21	22–29	22–70
	II BPD	21	22–29	≥30	>70
	III BPD	>21	≥30	-	-
	IIIA BPD	Death from respiratory causes between 14 days after birth and 36 weeks' PMA.			
2019 NRN criteria	1 BPD	Nasal cannula ≤ 2 L/min			
	2 BPD	Nasal cannula > 2 L/min, nCPAP, or NIPPV			
	3 BPD	IMV			

BPD, bronchopulmonary dysplasia; NCPAP, nasal continuous positive airway pressure; PMA, postmenstrual age; PPV, positive-pressure ventilation; IMV, invasive mechanical ventilation; NIPPV, noninvasive positive pressure ventilation.

The diagnostic criteria for adverse prognosis in the respiratory system were as followed (21): (1) hospitalization for respiratory reasons at ≥45 weeks' PMA (mean corrected age at discharge plus 2 standard deviations for very preterm infants in the last 10 years in our center), (2) use of supplemental oxygen, respiratory support, or respiratory monitoring (e.g., pulse oximeter or apnea monitor) at follow-up (3) tracheostomy placed any time before follow-up, or (4) rehospitalization for respiratory diseases such as acute bronchitis or pneumonia ≥2 times before the end of follow-up.

### Follow-up

All infants were followed up at 18 months' corrected age by specially trained physicians to assess the need for oxygen therapy or respiratory monitoring following initial discharge, or two or more hospitalizations for respiratory reasons before follow-up. All follow-up was completed by March 2025.

### Outcomes and group allocation

Based on the follow-up results, the preterm infants were divided into a respiratory adverse outcome group and a normal outcome group.

### Statistical analysis

The analysis was performed via R Studio 4.0.3. Normally distributed continuous variables are expressed as the mean ± standard deviation ($\bar{x}\pm s$) and compared between groups via independent-sample t tests. Non-normally distributed measurement data are presented as medians (interquartile ranges) [*M (Q1, Q3*)], and intergroup comparisons were performed via the Mann–Whitney *U* test. Categorical data are expressed as rates (percentages), and comparisons between groups were performed via the chi-square test or Fisher's exact test. The predictive value of different diagnostic criteria for prognosis was evaluated via receiver operating characteristic (ROC) curves, and differences in the area under the curve (AUC) were compared via the DeLong test. All the statistical tests were two-sided, and *P* < 0.05 was considered statistically significant.

### Machine learning model construction

Data preprocessing and the development and validation of the predictive model were performed via R Studio 4.0.3 and Python 3.9.10. In R Studio 4.0.3, the dataset was randomly divided into training (277 cases) and testing (120 cases) sets at a ratio of 7:3. Using the Python 3.9.15 with XGBoost 2.1.3 and scikit-learn 1.5.0 packages, an extreme gradient boosting (XGBoost) algorithm-based binary classification model was established for the prognostic prediction of BPD. The model was trained on the training cohort via 5-fold cross-validation, where the training data were partitioned into five equal subsets. Four subsets were iteratively used for model training, whereas the remaining subset served for validation across five cycles. During this process, model parameters were optimized on the basis of the receiver operating characteristic (ROC) curve and the area under the curve (AUC) to prevent overfitting. Finally, the model was validated on the testing dataset.

### Model performance evaluation

Overall, the performance of model was assessed in terms of accuracy, sensitivity, specificity and F1 score. The predictive value of the model was evaluated by receiver operating characteristic (ROC) curve analysis and the area under the curve (AUC). Additionally, to assess the utility of models for decision-making by quantifying the net benefit at different threshold probabilities, decision curve analysis (DCA) was conducted (22).

### Interpretability analysis

The SHapley Additive exPlanations (SHAP) Python package (version 0.46.0) was employed to perform interpretability analysis on the best-performing black-box model. The average of the absolute SHAP values of the selected feature parameters was defined as the importance of these parameters, and they were ranked accordingly. Additionally, a quantitative analysis of the main risk factors was performed on an individual feature basis.

## Results

### Characteristics of the study population

**Figure 1:**
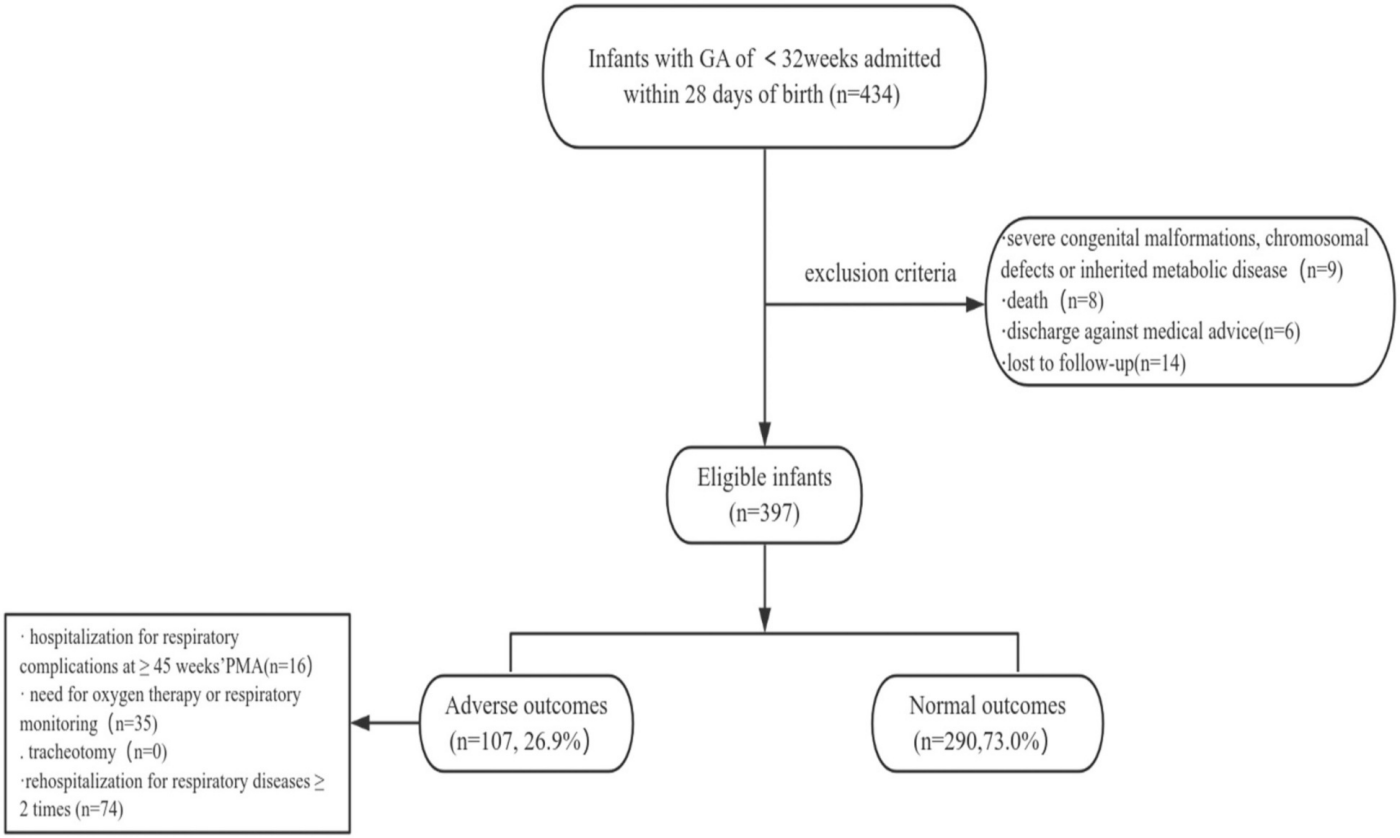
Flow diagram of the study population.

Between September 2021 and September 2023, a total of 434 preterm infants with a gestational age (GA) of <32 weeks were admitted within 28 days of birth to the Department of Neonatology of Children's Hospital Affiliated with Zhengzhou University. Excluded were 9 cases with severe congenital malformations, chromosomal abnormalities, or hereditary metabolic disorders; death occurred in 8 cases (7 deaths before 36 weeks PMA, 1 death after 36 weeks PMA primarily due to necrotizing enterocolitis); 6 cases were voluntarily discharged (4 prior to 36 weeks PMA, 2 after 36 weeks PMA); and 14 cases were lost to follow-up. Ultimately, 397 cases were included in the final analysis (Figure 1). A cohort of 397 preterm infants was analysed, and the gestational age and birth weight were 29.6 (28.4, 31.1) weeks and 1,270 (1,060, 1,500) g. The study population comprised 227 males (57.1%). According to the 2001 NICHD criteria, 263 cases (66.25%) of BPD were diagnosed, while the 2018 NICHD criteria and the 2019 NRN criteria identified 233 cases (58.69%). During the follow-up, 107 infants (26.9%) developed adverse respiratory outcomes. Specifically, 16 infants required ongoing hospitalization for respiratory complications at ≥45 weeks' PMA; 35 infants continued to need for oxygen therapy or pulse oximetry monitoring at initial discharge; 0 cases of tracheotomy were performed and 74 infants experienced respiratory disease-related rehospitalizations (≥2 episodes) prior to follow-up termination.

Comparison of the characteristics of preterm infants with different outcomes: Compared with the normal respiratory prognosis group, the proportion of early invasive ventilation, hemodynamically significant patent ductus arteriosus, and intraventricular hemorrhage of grade III were significantly higher in the adverse prognosis group (*P* < 0.05), whereas the proportions of early non-invasive ventilation, gestational age, birth weight, and weight at 36 weeks' PMA were significantly lower (*P* < 0.05). Additionally, Under the 2001 NICHD criteria for severe BPD, the 2018 NICHD criteria for grade II and III BPD, and the 2019 NRN criteria for grade 2 and 3 BPD, all demonstrated higher proportions of adverse respiratory outcomes compared to their respective normal outcomes (Table 2).

**Table 2:** Characteristics of preterm infants with different respiratory outcomes.

Variables	Normal outcomes	Adverse outcomes	*P-*value
	(*n* = 290)	(*n* = 107)	
Infant Profile			
Male (*N*, %)	165 (56.9)	62 (57.9)	0.942
GA(w), M(Q1, Q3)	30 (28.6, 31.2)	29.3 (27.5, 30.4)	<0.001
BW(g), M(Q1, Q3)	1,300 (1,100, 1,520)	1,200 (995, 1,440)	<0.001
Weight at 36 weeks' PMA (g), M(Q1,Q3)	2,050 (1,820, 2,230)	1,910 (1,680, 2,210)	0.023
SGA (*N*, %)	32 (11.0)	17 (15.9)	0.257
EUGR at 36 weeks' PMA (*N*, %)	184 (63.4)	75 (70.1)	0.265
Assisted reproduction (*N*, %)	19 (6.6)	14 (13.1)	0.059
Treatment with PS (*N*, %)	188 (64.8)	78 (72.9)	0.162
Treatment with caffeine (*N*, %)	189 (65.2)	69 (64.5)	0.993
Early invasive ventilation (*N*, %)	163 (56.2)	88 (82.2)	<0.001
Early non-invasive ventilation (*N*, %)	199 (68.6)	42 (39.3)	<0.001
hsPDA(*N*, %)	35 (12.1)	26 (24.3)	0.004
BPD-PH(*N*, %)	0 (0.0)	2 (1.9)	0.125
IVH (grade ≥III, N, %)	44 (15.2)	27 (25.2)	0.030
NEC (grade ≥II, N, %)	44 (15.2)	15 (14.0)	0.898
Sepsis(*N*,%)	140 (48.3)	56 (52.3)	0.545
Maternal Pregnancy Conditions			
HIP (*N*,%)	53 (18.3)	19 (17.8)	1
PROM (*N*,%)	101 (34.8)	28 (26.2)	0.13
GDM (*N*,%)	18 (6.2)	8 (7.5)	0.822
Eclampsia (*N*,%)	26 (9.0)	11 (10.3)	0.837
Prenata corticosteroids, (*N*,%)	202 (69.7)	73 (68.2)	0.879
Diagnostic criteria			
2001 NICHD criteria(%)			<0.001
Non BPD	111 (38.3)	23 (21.5)	
Mild BPD	29 (10.0)	2 (1.9)	
Moderate BPD	48 (16.6)	10 (9.3)	
Severe BPD	102 (35.2)	72 (67.3)	
2018 NICHD criteria(%)			<0.001
Non BPD	139 (47.9)	25 (23.4)	
I BPD	63 (21.7)	10 (9.3)	
II BPD	62 (21.4)	25 (23.4)	
III BPD	26 (9.0)	47 (43.9)	
2019 NRN criteria (%)			<0.001
Non BPD	139 (47.9)	25 (23.4)	
1 BPD	113 (39.0)	28 (26.2)	
2 BPD	29(10.0)	39(36.4)	
3 BPD	9(3.1)	15(14.0)	

SGA, small for gestational age; PMA, postmenstrual age; PS, pulmonary surfactant; GA, gestational age; BW, birth weight; EUGR, extrauterine growth restriction; hsPDA, hemodynamically significant patent ductus arteriosus; PH, pulmonary hypertension; IVH, intraventricular hemorrhage; NEC, neonatal necrotizing enterocolitis; HIP, hypertension in pregnancy; PROM, premature rupture of membranes; GDM, gestational diabetes mellitus; BPD, bronchopulmonary dysplasia.

**Model training and validation**: Comparative analysis of the prognostic validity of distinct diagnostic criteria in predicting infants' adverse respiratory outcomes.

**Figure 2:**
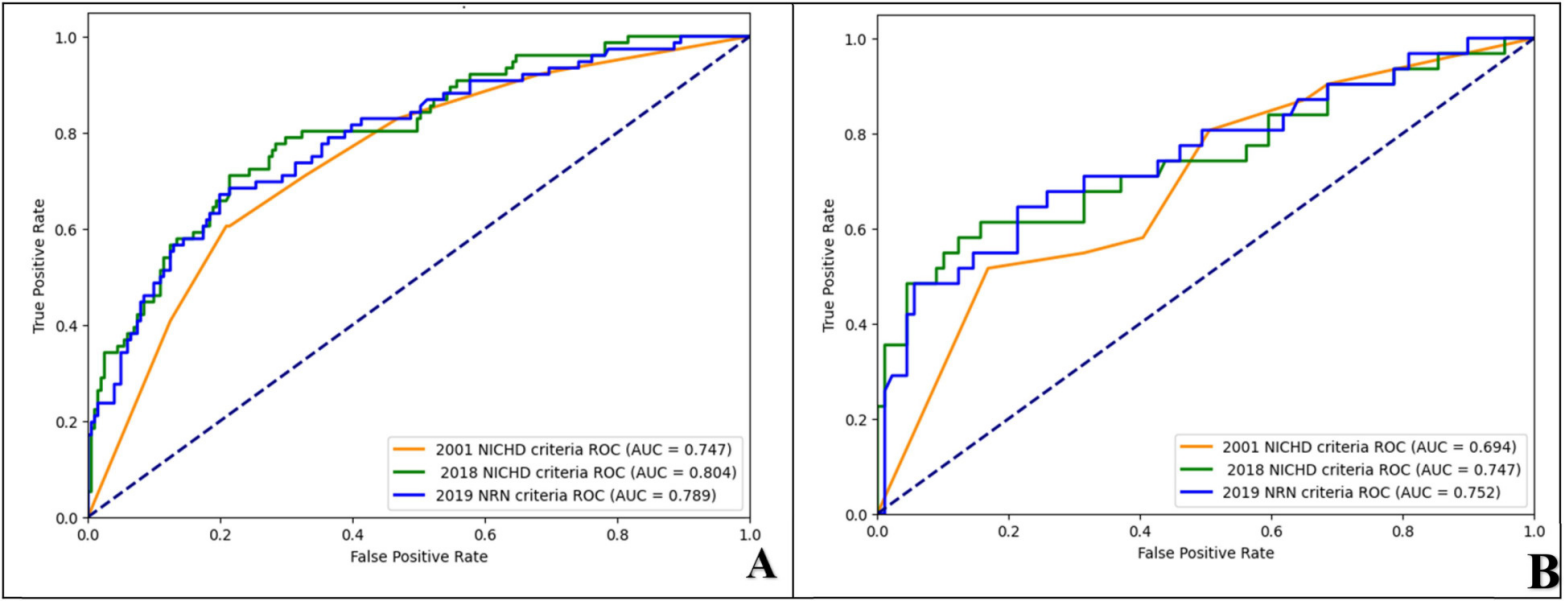
ROC curves of the prediction models for adverse respiratory outcomes in preterm infants under different BPD diagnostic criteria within the training and test sets. **(A)** training set; **(B)** test set.

**Figure 3:**
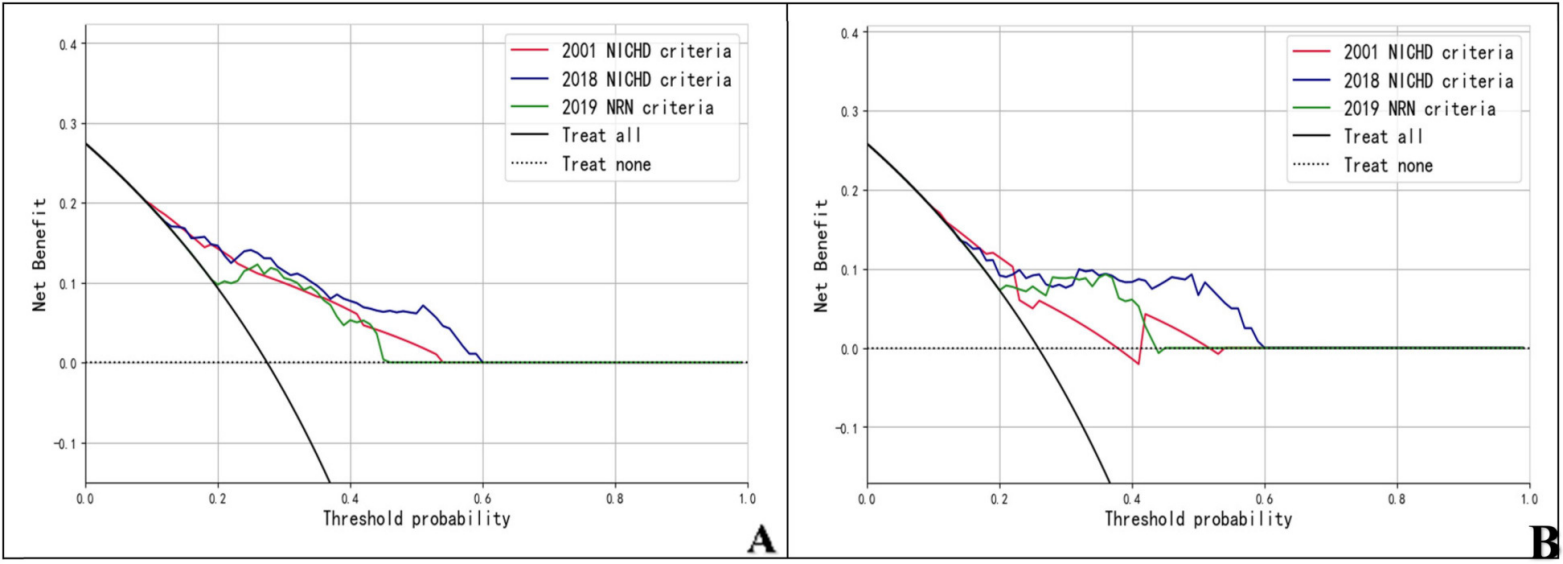
Decision curve analysis of models for adverse respiratory outcomes in preterm infants under different BPD diagnostic criteria in the training and test sets. **(A)** training set; **(B)** test set.

A total of 397 pediatric cases were randomly allocated into training and test sets at a 7:3 ratio, comprising 277 patients in the training set and 120 patients in the test set. No statistically significant differences were observed in the baseline characteristics between the training set and validation set (all *P* > 0.05; Table 3). Within the training cohort, 76 patients (27.4%) exhibited adverse respiratory outcomes, whereas the test set included 31 patients (25.8%) with adverse respiratory outcomes. By comparing the standardized mean differences in variables between the training and test datasets and calculating the corresponding values, the results in Table 4 show that the training and test sets have similar distributions across all variables, ensuring good comparability. Moreover, to further prevent overfitting, the built-in parameters of XGBoost were employed to control overfitting. ROC curves were constructed to evaluate the predictive performance of the three diagnostic criteria for adverse respiratory outcomes, as illustrated in Figure 2. In the training set, the AUC values of the prediction models based on the 2001 NICHD criteria, 2018 NICHD criteria, and 2019 NRN criteria were 0.747, 0.804, and 0.789, respectively, with accuracy of 0.740, 0.765, and 0.765, respectively (Table 5). In the test set, the AUC values of the prediction models based on the 2001 NICHD criteria, 2018 NICHD criteria, and 2019 NRN criteria were 0.694, 0.747, and 0.752, respectively, with accuracy of 0.750, 0.800, and 0.750, respectively (Table 5). Comparisons via the DeLong method reveled that, in both the training and test sets, the AUC values of the 2018 NICHD and 2019 NRN criteria were significantly higher than those of the 2001 NICHD criteria (training set: *Z* = −3.514 and −2.110, both *P* < 0.05; test set: *Z* = −2.137 and −2.199, both *P* < 0.05). However, no significant differences were found between the 2018 NICHD and 2019 NRN criteria in either the training set (*Z* = 0.863, *P* = 0.388) or the validation set (*Z* = −0.176, *P* = 0.861). Decision curve analysis (DCA) was performed to meet the practical needs of clinical decision-makers. The results revealed that, across most threshold probability ranges, the 2001 NICHD, 2018 NICHD and 2019 NRN criteria demonstrated favorable net benefits in both the training and test sets, suggesting acceptable clinical applicability (Figure 3).

**Table 3:** Characteristics of preterm infants with training and test sets.

Variables	Training sets	Test sets	*P-*value
	(*n* = 277)	(*n* = 120)	
Infant Profile			
Adverse outcomes (*N*, %)	76 (27.4)	31 (25.8)	0.836
Male (*N*, %)	154 (55.6)	73 (60.8)	0.391
GA(w), M(Q1,Q3)	29.5 (28.4, 31)	30 (28.4, 31.2)	0.574
BW(g), M(Q1, Q3)	1,270 (1,090, 1,500)	1,280 (1,057.5, 1,500)	0.835
Weight at 36 weeks' PMA (g), M(Q1,Q3)	2,030 (1,770, 2,230)	2,020 (1,800, 2,225)	0.962
SGA (*N*,%)	29 (10.5)	20 (16.7)	0.119
EUGR at 36 weeks' PMA (*N*,%)	180 (65.0)	79 (65.8)	0.961
Assisted reproduction (*N*,%)	20 (7.2)	13 (10.8)	0.317
Treatment with PS (*N*,%)	186 (67.1)	80 (66.7)	1
Treatment with caffeine (*N*,%)	174 (62.8)	84 (70.0)	0.206
Early invasive ventilation (*N*,%)	175 (63.2)	76 (63.3)	1
Early non-invasive ventilation (*N*,%)	172 (62.1)	69 (57.5)	0.454
hsPDA(*N*,%)	42 (15.2)	19 (15.8)	0.985
BPD-PH(*N*,%)	2 (0.7)	0 (0.0)	0.872
IVH (grade ≥III, N,%)	54 (19.5)	17 (14.2)	0.259
NEC (grade ≥II, N, %)	44 (15.9)	15 (12.5)	0.473
Sepsis(*N*, %)	137 (49.5)	59 (49.2)	1
Maternal Pregnancy Conditions			
HIP (*N*, %)	48 (17.3)	24 (20.0)	0.622
PROM (*N*, %)	95 (34.3)	34 (28.3)	0.295
GDM (*N*, %)	17 (6.1)	9 (7.5)	0.777
Eclampsia (*N*, %)	23 (8.3)	14 (11.7)	0.384
Prenata corticosteroids, (*N*, %)	189 (68.2)	86 (71.7)	0.573
Diagnostic criteria			
2001 NICHD criteria(%)			0.991
Non BPD	94 (33.9)	40 (33.3)	
Mild BPD	22 (7.9)	9 (7.5)	
Moderate BPD	41 (14.8)	17 (14.2)	
Severe BPD	120 (43.3)	54 (45.0)	
2018 NICHD criteria(%)			0.611
Non BPD	115 (41.5)	49 (40.8)	
I BPD	49 (17.7)	24 (20.0)	
II BPD	65 (23.5)	22 (18.3)	
III BPD	48 (17.3)	25 (20.8)	
2019 NRN criteria (%)			0.386
Non BPD	115 (41.5)	49 (40.8)	
1 BPD	100 (36.1)	41 (34.2)	
2 BPD	49 (17.7)	19 (15.8)	
3 BPD	13 (4.7)	11 (9.2)	

SGA, small for gestational age; PMA, postmenstrual age; PS, pulmonary surfactant; GA, gestational age; BW, birth weight; EUGR, extrauterine growth restriction; hsPDA, hemodynamically significant patent ductus arteriosus; PH, pulmonary hypertension; IVH, intraventricular hemorrhage; NEC, neonatal necrotizing enterocolitis; HIP, hypertension in pregnancy; PROM, premature rupture of membranes; GDM, gestational diabetes mellitus; BPD, bronchopulmonary dysplasia.

**Table 4:** Comparison of standardized mean differences in variables between training and test sets.

Variables	Training sets	Test sets	*P-*value	Test SMD
	(*n* = 277)	(*n* = 120)		
Infant Profile				
Adverse outcomes[mean (SD)]	0.27 (0.45)	0.26 (0.44)	0.742	0.036
Male [mean (SD)]	0.44 (0.50)	0.39 (0.49)	0.334	0.106
GA [mean (SD)]	29.49 (1.61)	29.59 (1.69)	0.562	0.063
BW(g) [mean (SD)]	1,297.96 (304.07)	1,308.29 (348.36)	0.766	0.032
Weight in PMA36w [mean (SD)]	2,002.61 (335.47)	2,009.92 (364.28)	0.846	0.021
SGA [mean (SD)]	0.10 (0.31)	0.17 (0.37)	0.085	0.181
EUGR at 36 weeks' PMA [mean (SD)]	0.65 (0.48)	0.66 (0.48)	0.87	0.018
Assisted reproduction [mean (SD)]	0.07 (0.26)	0.11 (0.31)	0.232	0.126
Treatment with PS [mean (SD)]	0.67 (0.47)	0.67 (0.47)	0.926	0.010
Treatment with caffeine [mean (SD)]	0.63 (0.48)	0.70 (0.46)	0.169	0.152
Early invasive ventilation [mean (SD)]	0.63 (0.48)	0.63 (0.48)	0.976	0.003
Early non-invasive ventilation[mean (SD)]	0.62 (0.49)	0.58 (0.50)	0.391	0.094
hsPDA [mean (SD)]	0.15 (0.36)	0.16 (0.37)	0.865	0.018
BPD-PH [mean (SD)]	0.01 (0.08)	0.00 (0.00)	0.352	0.120
IVH [grade ≥III, mean (SD)]	0.19 (0.40)	0.14 (0.35)	0.204	0.142
NEC [grade ≥II, mean (SD)]	0.16 (0.37)	0.12 (0.33)	0.385	0.097
Sepsis [mean (SD)]	0.49 (0.50)	0.49 (0.50)	0.958	0.006
Maternal Pregnancy Conditions				
HIP [mean (SD)]	0.17 (0.38)	0.20 (0.40)	0.527	0.068
PROM [mean (SD)]	0.34 (0.48)	0.28 (0.45)	0.245	0.128
GDM [mean (SD)]	0.06 (0.24)	0.07 (0.26)	0.615	0.054
Eclampsia [mean (SD)]	0.08 (0.28)	0.12 (0.32)	0.291	0.112
Prenatal corticosteroids [mean (SD)]	0.68 (0.47)	0.72 (0.45)	0.497	0.075
Diagnostic criteria				
2001 NICHD criteria [mean (SD)]				
Non BPD	0.34 (0.47)	0.33 (0.47)	0.908	0.013
Mild BPD	0.08 (0.27)	0.07 (0.26)	0.880	0.017
Moderate BPD	0.15 (0.36)	0.14 (0.35)	0.870	0.018
Severe BPD	0.43 (0.50)	0.45 (0.50)	0.758	0.034
2018 NICHD criteria[mean (SD)]				
Non BPD	0.42 (0.49)	0.41 (0.49)	0.899	0.014
I BPD	0.18 (0.38)	0.20 (0.40)	0.586	0.059
II BPD	0.23 (0.42)	0.18 (0.39)	0.257	0.126
III BPD	0.17 (0.38)	0.21 (0.41)	0.409	0.089
2019 NRN criteria [mean (SD)]				
Non BPD	0.42 (0.49)	0.41 (0.49)	0.899	0.014
1 BPD	0.36 (0.48)	0.34 (0.48)	0.712	0.040
2 BPD	0.18 (0.38)	0.16 (0.37)	0.653	0.050
3 BPD	0.05 (0.21)	0.09 (0.29)	0.086	0.176

SGA, small for gestational age; PMA, postmenstrual age; PS, pulmonary surfactant; GA, gestational age; BW, birth weight; EUGR, extrauterine growth restriction; hsPDA, hemodynamically significant patent ductus arteriosus; PH, pulmonary hypertension; IVH, intraventricular hemorrhage; NEC, neonatal necrotizing enterocolitis; HIP, hypertension in pregnancy; PROM, premature rupture of membranes; GDM, gestational diabetes mellitus; BPD, bronchopulmonary dysplasia.

**Table 5:** Predictive performance metrics of models for adverse respiratory outcomes in preterm infants under different BPD diagnostic criteria in the training and test sets.

Different criteria	Training set						Test set					
	Accuracy	F1-score	Sensitivity	Specificity	PPV	NPV	Accuracy	F1-score	Sensitivity	Specificity	PPV	NPV
2001 NICHD criteria	0.740	0.561	0.605	0.791	0.523	0.841	0.750	0.516	0.516	0.831	0.516	0.831
2018 NICHD criteria	0.765	0.624	0.711	0.786	0.577	0.878	0.800	0.600	0.581	0.876	0.621	0.857
2019 NRN criteria	0.765	0.611	0.671	0.801	0.560	0.866	0.750	0.570	0.645	0.787	0.513	0.864

PPV, positive predictive value; NPV, negative predictive value.

### Feaure importance assessed by SHAP values

**Figure 4:**
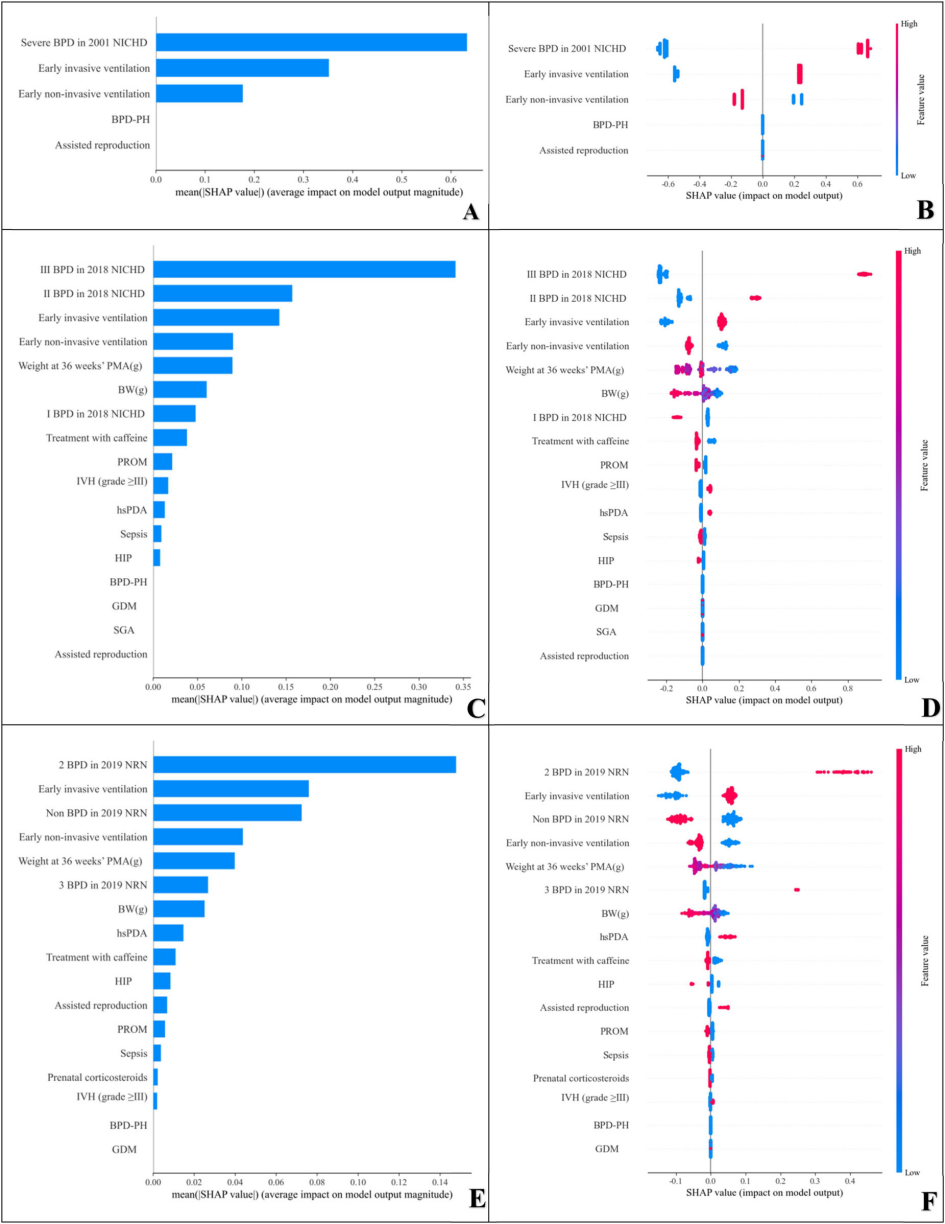
Global model explanation: summary bar plot and summary point plot in SHAP analysis. SHAP, the SHapley additive exPlanation; PH, pulmonary hypertension; PMA, postmenstrual age; BW, birth weight; PROM, premature rupture of membranes; IVH, intraventricular hemorrhage; hsPDA, hemodynamically significant patent ductus arteriosus; HIP, hypertension in pregnancy; SGA, small for gestational age; GDM, gestational diabetes mellitus; SHAP, summary bar plot. This plot displays the weights of variable importance on the basis of Shapley values. SHAP, summary point plot. Each point represents a Shapley value for a specific patient and feature. The color of the points represents actual feature values, with red dots representing high-risk values and blue dots indicating low-risk values. The higher the SHAP value of a feature is, the greater the likelihood of an adverse respiratory outcome. **(A)** SHAP summary bar plot of the 2001 NICHD criteria; **(B)** SHAP summary point plot of the 2001 NICHD criteria; **(C)** SHAP summary bar plot of the 2018 NICHD criteria; **(D)** SHAP summary point plot of the 2018 NICHD criteria; **(E)** SHAP summary bar plot of the 2019 NRN criteria; **(F)** SHAP summary point plot of the 2019 NRN criteria.

The feature parameter importance ranking results demonstrated that under the 2001 NICHD criteria, severe BPD presented the greatest predictive value for respiratory outcomes in preterm infants, followed by early invasive ventilation and early non-invasive ventilation (Figure 4A). Further analysis revealed that severe BPD and early invasive ventilation were positively correlated with adverse respiratory outcomes in preterm infants. That is, preterm infants with severe BPD and early invasive ventilation in the model had a greater likelihood of experiencing adverse respiratory outcomes. Conversely, early non-invasive ventilation was negatively correlated with adverse respiratory outcomes in preterm infants (Figure 4B). It should be noted that in the SHAP analysis presented in Figures 4A,B, features are ordered by the mean of absolute SHAP values, which is a conventional method for evaluating feature importance. This method emphasizes the features that have the most significant impact on model predictions. Figures 4A,B specifically highlight the top five features with the highest SHAP values to underscore the primary drivers of the model's outcomes. The remaining features are not displayed in Figures 4A,B due to their relatively low mean SHAP values. According to 2018 NICHD criteria, Grade III BPD had the greatest predictive value for respiratory prognosis in preterm infants, followed by early invasive ventilation and Grade II BPD (Figure 4C). Further analysis revealed that Grade III BPD, early invasive ventilation, and Grade II BPD were positively correlated with adverse respiratory outcomes in preterm infants, indicating that preterm infants with these conditions in the model presented an increased likelihood of developing adverse respiratory outcomes (Figure 4D). According to the 2019 NRN criteria, grade 2 BPD had the strongest predictive value for adverse respiratory outcomes in preterm infants, followed by early invasive ventilation and non-BPD (Figure 4E). Subsequent analyses revealed that grade 2 BPD and early invasive ventilation were positively correlated with adverse respiratory outcomes in preterm infants (Figure 4F).

## Discussion

The diagnostic criteria for BPD in preterm infants were initially proposed by Northway (23) in 1967 and have been subsequently revised with enhanced understanding of its pathophysiology and advances in clinical respiratory care techniques. The diagnostic criteria for BPD in preterm infants, proposed by NICHD in 2001 (19), were widely used clinically and rflected the pathological changes of “new” BPD. However, since the beginning of this century, noninvasive ventilation has continued to progress, and heated and humidified high-flow oxygen therapy has been widely used in clinical practice, with the appropriate oxygen saturation range for preterm infants also changing (24). Therefore, in 2018, NICHD (20) revised the 2001 criteria (19)^,^ preterm infants born at less than 32 weeks of gestation who required continuous oxygen therapy for more than 28 days after birth were classified based on their FiO2 and respiratory support at a PMA 36 weeks, with a comprehensive and detailed classification. In 2019, the NRN (21) proposed its 2019 BPD criteria on the basis of extensive evidence base. This criteria classifies BPD solely according to the respiratory support required at 36 weeks'PMA, making it straightforward for clinical implementation.

Our preliminary studies (25) demonstrated that the widely used 2001 NICHD criteria definition failed to consider advancements in respiratory support and expanded the diagnosis of BPD, resulting in an underestimation of mortality for severe BPD patients. The 2018 NICHD criteria and 2019 NRN criteria show high overall diagnostic consistency but weak consistency in severity grading. This study applied machine learning, enabling computers to analyze and learn patterns from massive datasets to predict or make decisions in new scenarios. This method emphasizes prediction accuracy and can uncover patterns in multidimensional datasets (18). The datasets were split into training and validation sets in a 7:3 ratio to evaluate the predictive value of three diagnostic criteria for respiratory outcomes. The findings revealed that the 2018 and 2019 criteria have similar and superior predictive performance for long-term prognosis compared to the 2001 NICHD criteria. This is consistent with the results of Pérez-Tarazona et al. (26) and our earlier studies using traditional regression methods (11). However, the study by Pérez-Tarazona (26) differs in population characteristics as it only examined long-term prognosis of BPD preterms, whereas our study included all preterms meeting the inclusion criteria.

The 2018 NICHD's BPD diagnostic criteria (20) were based on expert consensus, with limited clinical validation of their prognostic value, which is yet to be validated in a large neonatal population (27). This study found that the criteria achieved 76.5% accuracy in predicting respiratory outcomes. The 2019 NRN BPD criteria (21), developed using evidence-based medicine, were validated through rigorous statistics in a large multicenter population. Compared to 17 previous diagnostic criteria, they can accurately predict adverse outcomes (death, respiratory and neurological outcomes) in 81% of infants at 18–26 months of age. The predictive accuracy of this study in the training cohort for respiratory outcomes using the 2019 NRN criteria was observed to be 76.5%. This discrepancy may be attributed to the following factors: First, the included population exhibited a median gestational age of 29.6 (28.4, 31.1) weeks, whereas the 2019 NRN cohort primarily comprised extremely preterm infants with gestational age <27 weeks (89% of cases). Second, this study exclusively focused on respiratory outcomes and did not account for mortality or neurological outcomes.

In this study, adverse respiratory outcomes were defined with reference to the criteria established by Jensen et al. (21), where hospitalization due to respiratory conditions at PMA of 50 weeks was included as one criterion for poor respiratory prognosis. The selection of 45 weeks PMA as the time point in this study was based on epidemiological data showing that 45 weeks represents the mean corrected age plus two standard deviations of the initial discharge timing for extremely preterm infants in our institution over the past decade.

Additionally, we employed the SHAP method to interpret the model. SHAP is a *post hoc* interpretation framework for black-box models based on Shapley values from game theory, which can help researchers understand the impact of features on predicted outcomes (18).The findings demonstrated that among multiple high-risk factors, the severity of BPD constituted the most significant determinant influencing respiratory outcomes in preterm infants, followed by early invasive ventilation. Severe BPD under the 2001 NICHD criteria, grade 3 BPD per the 2018 NICHD criteria, and grade 2 BPD based on the 2019 NRN criteria all ranked first in predictive significance. Notably, grade 3 BPD defined by the 2019 NRN criteria exhibited a comparatively lower ranking in contribution weight, potentially attributable to its limited sample size of 24 cases (6.0%) in this cohort. In the SHAP variable scatter plot, each point corresponds to a sample. According to the 2019 NRN criteria for grade 3 BPD, the cohort was restricted to preterm infants requiring invasive mechanical ventilation, compared to the 2018 criteria excluded infants managed with non-invasive ventilation requiring oxygen flow rates ≥3 L/min combined with FiO₂ ≥ 30%. Future studies with larger sample sizes are warranted to validate these comparisons. Hwang et al. reported (28) that under the 2018 NICHD and 2019 NICHD definitions, higher BPD grades—particularly Grade 3—were strongly associated with worse outcomes. Specifically, for rehospitalization, the adjusted odds ratio for Grade 3 BPD under the 2019 NICHD definition was 5.72 (95% CI 1.37–23.9). Many studies have demonstrated that, regardless of the BPD diagnostic criteria used, preterm infants with grade II or III BPD are more prone to adverse respiratory outcomes such as lower respiratory tract infections and rehospitalization in the long term compared to those with grade I BPD (29, 30). This further underscores the significant link between BPD severity and adverse respiratory prognosis.

In the SHAP analysis, early invasive ventilation ranked second in contributing to adverse respiratory outcomes in preterm infants and showed a positive correlation, which is consistent with previous studies. Invasive mechanical ventilation during the early postnatal period has been shown to contribute to ventilator-associated lung injury in preterm infants, significantly increasing the incidence of BPD (31) and representing a risk factor for adverse respiratory outcomes. A cohort study of 3,343 extremely low - birth—weight infants showed that in survivors, early and prolonged invasive ventilation is an important factor increasing BPD incidence and likelihood of adverse outcomes like ongoing oxygen therapy at discharge (32). In this study, under the three diagnostic criteria, early non-invasive ventilation was negatively correlated with adverse respiratory outcomes in preterm infants and was a protective factor. Similarly, Kaltsogianni-Ourania et al. (33) studies have shown that compared with invasive mechanical ventilation, the early application of non-invasive respiratory support not only reduces the incidence of BPD but also decreases the rate of respiratory morbidity at a corrected age of 18–22 months. The umbrella systematic review by Abiramalath et al. (31) provides high-quality evidence demonstrating that, for preterm infants with a gestational age of less than 30 weeks, early application of non-invasive continuous positive airway pressure combined with minimally invasive surfactant administration in the delivery room is associated with reduced risks of adverse outcomes. These findings are consistent with our research, suggesting that selecting non-invasive respiratory support early after birth in very preterm infants is an important measure to reduce the incidence of long-term respiratory morbidity.

There were some limitations in our study. First, this was a single-center study with a relatively small sample size of extremely preterm infants born before 28 weeks of gestation (77 cases, 19.4%), a population at higher risk for BPD. Secondly, the absence of an external validation cohort may limit the generalizability of our findings. Future multicenter studies focusing on infants born at <28 weeks' gestation are warranted, incorporating external validation to better evaluate the predictive value of different diagnostic criteria for prognostic outcomes. Finally, note that SHAP values are calculated based on the assumption of predictor independence. However, in real-world data, certain predictors such as those related to BPD may be correlated, which can affect the accuracy and interpretability of SHAP values. Aas et al. (34) introduced an enhanced Kernel SHAP method that can handle feature dependencies and provide a more accurate interpretation of feature importance. We plan to further explore and apply such methods in future research to enhance the precision of model prediction explanations.

## Conclusion

In this study, we employed interpretable machine learning approaches to compare and validate the predictive value of three diagnostic criteria for respiratory outcomes in preterm infants. The results demonstrated that the 2018 NICHD criteria and 2019 NRN criteria, which provide more cautious and rigorous diagnoses through systematic classification of respiratory support modes, demonstrate better predictive value for long-term adverse respiratory outcomes in preterm infants in both the training and validation cohorts. They exhibited greater accuracy in long-term follow-up of respiratory outcomes. These findings are consistent with those of prior studies by logistic regression methods, ensuring the reliability and generalizability of the results. This study provides clinicians with appropriate diagnostic and grading criteria, and lays a theoretical foundation for the development of future diagnostic standards.

## Data Availability

The raw data supporting the conclusions of this article will be made available by the authors, without undue reservation.
